# Analysis of Spatial and Temporal Distribution of Purinergic P2 Receptors in the Mouse Hippocampus

**DOI:** 10.3390/ijms22158078

**Published:** 2021-07-28

**Authors:** Julian Lommen, Julika Detken, Katharina Harr, Charlotte von Gall, Amira A. H. Ali

**Affiliations:** Institute of Anatomy II, Medical Faculty and University Hospital Düsseldorf, Heinrich-Heine-University, Universitätsstrasse 1, 40225 Düsseldorf, Germany; julian.lommen@uni-duesseldorf.de (J.L.); julika.d@web.de (J.D.); Katharina.Harr@web.de (K.H.); amira.ali@med.uni-duesseldorf.de (A.A.H.A.)

**Keywords:** purinergic receptors, hippocampus, mouse brain, time domain, circadian, diurnal, chronobiology

## Abstract

ATP and other nucleotides are important glio-/neurotransmitters in the central nervous system. They bind to purinergic P2X and P2Y receptors that are ubiquitously expressed in various brain regions modulating various physiological and pathophysiological processes. P2X receptors are ligand-gated ion channels mediating excitatory postsynaptic responses whereas P2Y receptors are G protein-coupled receptors mediating slow synaptic transmission. A variety of P2X and P2Y subtypes with distinct neuroanatomical localization provide the basis for a high diversity in their function. There is increasing evidence that P2 receptor signaling plays a prominent role in learning and memory and thus, in hippocampal neuronal plasticity. Learning and memory are time-of-day-dependent. Moreover, extracellular ATP shows a diurnal rhythm in rodents. However, it is not known whether P2 receptors have a temporal variation in the hippocampus. This study provides a detailed systematic analysis on spatial and temporal distribution of P2 in the mouse hippocampus. We found distinct spatial and temporal distribution patterns of the P2 receptors in different hippocampal layers. The temporal distribution of P2 receptors can be segregated into two large time domains, the early to mid-day and the mid to late night. This study provides an important basis for understanding dynamic P2 purinergic signaling in the hippocampal glia/neuronal network.

## 1. Introduction

Purinergic P2 receptors are an integral part of cell-to-cell signaling processes within the mammalian central (CNS) and peripheral (PNS) nervous system [1,2]. P2 receptors can be divided into two classes: the ligand-gated ion channel P2X and the G-protein coupled P2Y which are further divided into the subclasses P2X1–7 and P2Y_1,2,4,6,11–14_ [3,4]. Most of them are predominantly activated by nucleotide signaling molecules, such as adenosine 5′-triphosphate (ATP) and its analogues [5]. P2 receptor inactivation is usually facilitated by ectoenzymatic breakdown of ATP [6]. ATP is released from astrocytes into the synaptic cleft via hemichannels or vesicular exocytosis where it can act on neuronal pre- or postsynaptic P2 receptors [7,8,9]. As ATP is an important transmitter and modulator in intercellular communication among neurons and glia cells [10], the biological relevance of P2 receptors in the brain is deemed high. The function of P2 receptor signaling can be differentiated in short-term effects such as transmission, modulation, and inflammation, mostly mediated by P2X [11], and long-term effects such as cell proliferation, development, regeneration, mostly mediated by P2Y [12,13,14]. Within the last two decades, there has been intensive scientific research in the field of P2 receptor signaling within the healthy and diseased brain and drugs targeted to P2 receptors have beneficial effects on a variety of neurological diseases [15]. Distribution and density of P2 receptor expression varies immensely between different brain regions [16,17]. Importantly, to fully understand P2 receptor signaling not only spatial but also temporal distribution should be considered [18]. We could show earlier, that P2 receptors show a time-of-day-dependent variation in the mouse suprachiasmatic nucleus (SCN) [19], the circadian rhythm generator [20,21,22]. Here, we focus on the spatial and temporal distribution of various P2 receptors in the hippocampus, the brain region of fundamental importance for learning and memory [23], processes which are strongly controlled by the circadian system [24]. Neocortical projections reach the entorhinal cortex, the source of the perforant path, which projects to all subregions of the hippocampus. Perforant path projections terminate at the stratum moleculare of the cornu ammonis (CA) 1 and the dentate gyrus (DG). The DG granule cells give rise to the mossy fiber pathway, which targets the stratum lucidum of the CA3. The CA3 Schaffer collaterals project primarily to the stratum radiatum of the CA1. The axons of the CA1 pyramidal neurons form the output paths to the subiculum and to subcortical regions [25,26]. The hippocampus also receives a variety of subcortical inputs e.g., from the amygdala, cholinergic forebrain nuclei such as the septal nuclei, hypothalamus and brain stem such as the raphe nuclei. Projections from the septal nuclei and from the raphe nuclei terminate primarily at the stratum oriens and lacunosum of the CA1, respectively [27]. Various P2 receptors have been detected in the rodent hippocampus such as P2X2 [28,29,30], P2X3 [31,32], P2X4 and P2X7 [33] as well as P2Y_1_ and P2Y_11–13_ [34,35,36]. Moreover, P2X_1–2_ and P2Y_1_ are reported to be involved in learning and memory processes [37,38,39]. However, little is known about the spatial and temporal distribution of P2 receptors within the hippocampal subregions. Therefore, in this study we analyzed the distribution of P2X1-7, P2Y_1,2_, P2Y_4_, P2Y_6_ and P2Y_11–14_–immunoreaction in layers of the hippocampus during the 24 h cycle. The description of distinct spatial and temporal distribution patterns provide an important basis for better understanding dynamic P2 receptor signaling in the hippocampus and thus for developing new therapeutic strategies against impairments of learning and memory. 

## 2. Results

### 2.1. Spatial Distribution of P2-IR in the Hippocampus

Spatial distribution of P2 receptors was assessed based on pseudocolor images of IR in hippocampal layers of mice sacrificed during the middle of the light/inactive phase (ZT06). 

#### 2.1.1. Spatial Distribution of P2X-IR 

**Figure 1:**
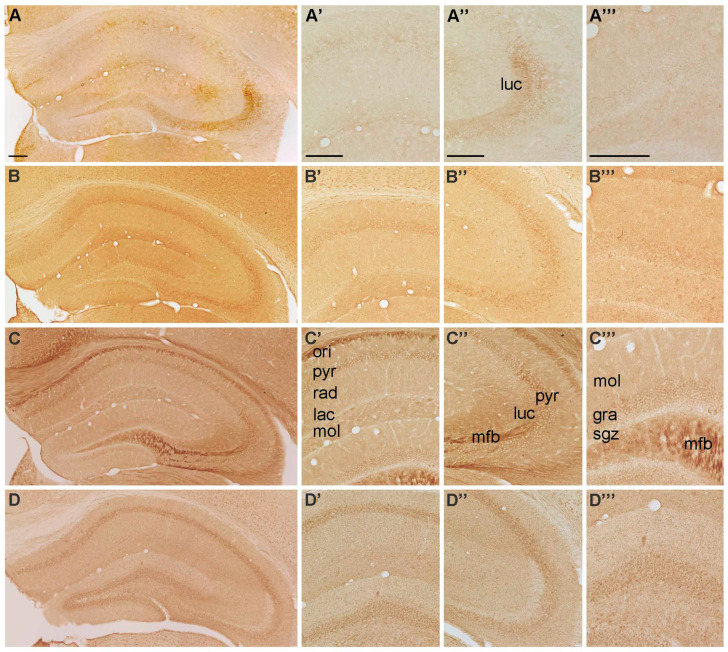
Representative microphotographs of (**A**) P2X1-, (**B**) P2X2-, (**C**) P2X3 and (**D**) P2X4-IR in the mouse hippocampus. At higher magnification **(A’)** P2X1-IR in cornu ammonis (CA) 1, (**A’’**) P2X1-IR in cornu ammonis (CA) 3, (**A’’’**) P2X1-IR in dentate gyrus, (**B’**) P2X2-IR in cornu ammonis (CA) 1, (**B’’**) P2X2-IR in cornu ammonis (CA) 3, (**B’’’**) P2X2-IR in dentate gyrus, (**C’**) P2X3-IR in cornu ammonis (CA) 1, **(C’’**) P2X3-IR in cornu ammonis (CA) 3, (**C’’’**) P2X3-IR in dentate gyrus, (**D’**) P2X4-IR in cornu ammonis (CA) 1, (**D’’**) P2X4-IR in cornu ammonis (CA) 3 and (**D’’’**) P2X4-IR in dentate gyrus, respectively. Abbreviations: ori, stratum oriens, pyr, stratum pyramidale, rad, stratum radiatum; lac, stratum lacunosum; mol, stratum moleculare; luc, stratum lucidum; mfb, mossy fiber bundle; gra, stratum granulare, sgz, subgranular zone. Scale bars = 200 µm.

**Figure 2:**
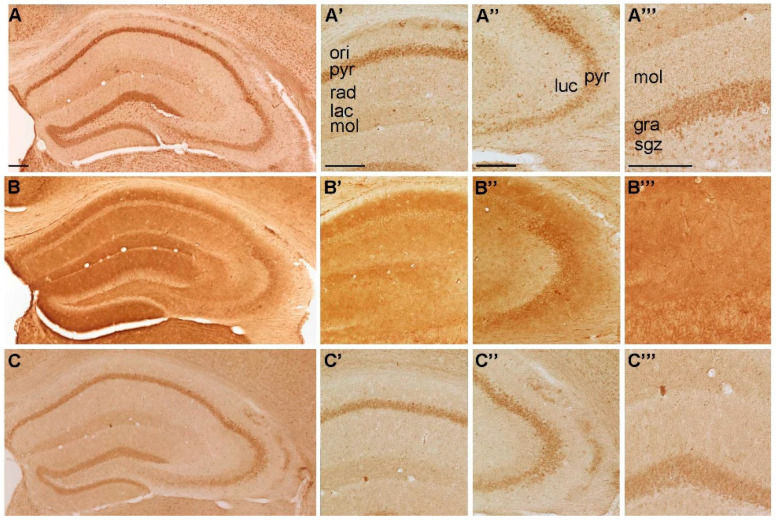
Representative microphotographs of (**A**) P2X5-, (**B**) P2X6-, and (**C**) P2X7-IR in the mouse hippocampus. At higher magnification (**A’**) P2X5-IR in cornu ammonis (CA) 1, **(A’’)** P2X5-IR in cornu ammonis (CA) 3, (**A’’’**) P2X5-IR in dentate gyrus, (**B’**) P2X6-IR in cornu ammonis (CA) 1, (**B’’**) P2X6-IR in cornu ammonis (CA) 3, (**B’’’**) P2X6-IR in dentate gyrus, (**C’**) P2X7-IR in cornu ammonis (CA) 1, (**C’’**) P2X7-IR in cornu ammonis (CA) 3 and **(C’’’)** P2X7-IR in dentate gyrus, respectively. Abbreviations: ori, stratum oriens, pyr, stratum pyramidale, rad, stratum radiatum; lac, stratum lacunosum; mol, stratum moleculare; luc, stratum lucidum; gra, stratum granulare, sgz, subgranular zone. Scale bars = 200 µm.

The overall IR of P2X1 in the hippocampus was comparatively weak. A strong P2X1-IR was only present in the stratum lucidum and molecular cell layer of the CA3 region (Figure 1A, Supplementary Figure S2A). P2X2-IR was moderate in the molecular cell layers of DG and CA1, granule cell layer of DG, and pyramidal cell layers of CA1 and CA3 (Figure 1B, Supplementary Figure S2B). A very strong P2X3-IR was present in the mossy fibers which contain zinc and can be visualized by Timm staining (Supplementary Figure S1) as well as in the stratum oriens of the CA3 and CA1 (Figure 1C, Supplementary Figure S2C). A moderate P2X4-IR (Figure 1D, Supplementary Figure S1D), P2X7-IR (Figure 2C, Supplementary. Figure S2G) and a strong P2X5-IR (Figure 2A, Supplementary Figure S2E) were present in the granule cell layer of the dentate gyrus and the pyramidal cell layers of the CA1 and CA3, respectively. P2X6-IR was strong in stratum moleculare of the DG, in pyramidal cell layer of CA3, as well as in stratum radiatum, stratum oriens and statum moleculare of the CA1 region (Figure 2B, Supplementary Figure S2F). P2X6-IR was moderate in the hilus of DG, in stratum lacunosum and stratum pyramidale of CA1 as well as in the stratum moleculare of the CA3 region (Figure 2B, Supplementary Figure S1F). 

#### 2.1.2. Spatial Distribution of P2Y-IR 

**Figure 3:**
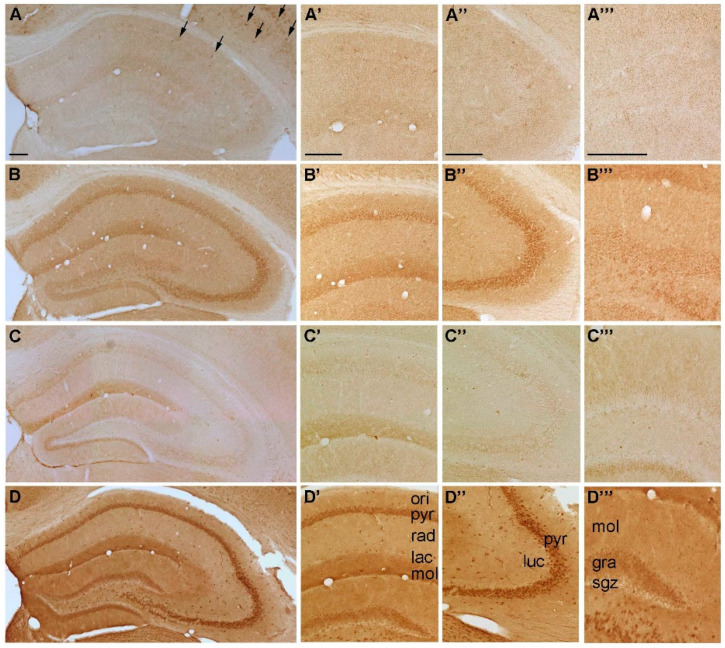
Representative microphotographs of (**A**) P2Y_1_-, (**B**) P2Y_2_-, (**C**) P2Y_4_ and (**D**) P2Y_6_-IR in the mouse hippocampus. At higher magnification (**A’**) P2Y1-IR in cornu ammonis (CA) 1, (**A’’**) P2Y1-IR in cornu ammonis (CA) 3, (**A’’’**) P2Y1-IR in dentate gyrus, (**B’**) P2Y2-IR in cornu ammonis (CA) 1, (**B’’**) P2Y2-IR in cornu ammonis (CA) 3, (**B’’’**) P2Y2-IR in dentate gyrus, (**C’**) P2Y4-IR in cornu ammonis (CA) 1, (**C’’**) P2Y4-IR in cornu ammonis (CA) 3, (**C’’’**) P2Y4-IR in dentate gyrus, (**D’**) P2Y6-IR in cornu ammonis (CA) 1, (**D’’**) P2Y6-IR in cornu ammonis (CA) 3 and (**D’’’**) P2Y6-IR in dentate gyrus, respectively. Abbreviations: ori, stratum oriens, pyr, stratum pyramidale, rad, stratum radiatum; lac, stratum lacunosum; mol, stratum moleculare; luc, stratum lucidum; gra, stratum granulare, sgz, subgranular zone. Arrows indicate cells with interneuron-like morphology. Scale bars = 200 µm.

**Figure 4:**
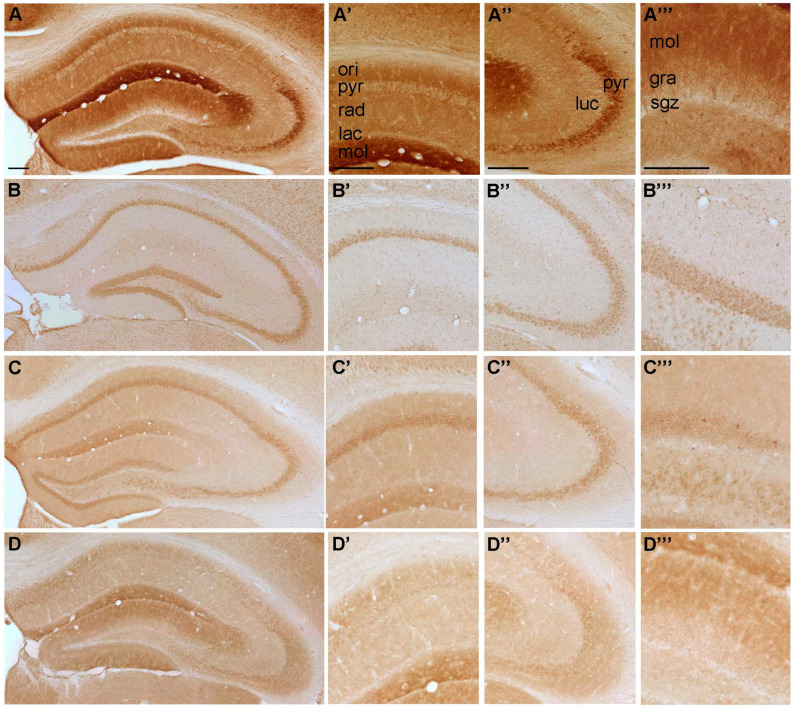
Representative microphotographs of (**A**) P2Y_11_-, (**B**) P2Y_12_-, (**C**) P2Y_13_ and (**D**) P2Y_14_-IR in the mouse hippocampus. At higher magnification (**A’**) P2Y11-IR in cornu ammonis (CA) 1, (**A’’**) P2Y11-IR in cornu ammonis (CA) 3, (**A’’’**) P2Y11-IR in dentate gyrus, (**B’**) P2Y12-IR in cornu ammonis (CA) 1, (**B’’**) P2Y12-IR in cornu ammonis (CA) 3, (**B’’’**) P2Y12-IR in dentate gyrus, (**C’**) P2Y13-IR in cornu ammonis (CA) 1, (**C’’**) P2Y13-IR in cornu ammonis (CA) 3, (**C’’’**) P2Y13-IR in dentate gyrus, (**D’**) P2Y14-IR in cornu ammonis (CA) 1, (**D’’**) P2Y14-IR in cornu ammonis (CA) 3 and (**D’’’**) P2Y14-IR in dentate gyrus, respectively. Abbreviations: ori, stratum oriens, pyr, stratum pyramidale, rad, stratum radiatum; lac, stratum lacunosum; mol, stratum moleculare; luc, stratum lucidum; gra, stratum granulare, sgz, subgranular zone. Scale bars = 200 µm.

The overall IR of P2Y_1_ was weak in the entire hippocampus (Figure 3A, Supplementary Figure S3A). The P2Y_2_-IR was moderate in the molecular cell layer of the CA1 region, in the granule cell layer (DG) and the pyramidal cell layer of CA1 and strong in the pyramidal cell layer of the CA3 (Figure 3B, Supplementary Figure S3B). Single cells in the hilus showed a moderate P2Y_2_-IR. P2Y_4_-IR in the granule cell layer (DG) as well as in the molecular cell layer of CA1 (Figure 3C, Supplementary Figure S3C). P2Y_6_-IR was strong in the granule cell layer (DG) and the molecular cell and pyramidal cell layer of the CA1 region and very strong in the pyramidal cell layer of CA3 (Figure 3D, Supplementary Figure S3D). P2Y_11_-IR was weak in granule cell layer of DG, pyramidal cell layer and stratum lacunosum of the CA1 as well as in stratum lucidum of CA3, moderate in stratum radiatum of CA1, strong in stratum moleculare of DG and pyramidal cell layer of CA3, and very strong in molecular cell layer of CA1 (Figure 4A, Supplementary Figure S3E and Table S2). P2Y_12_-IR was moderate in the granule (DG) and pyramidal (CA1 and CA3) cell layers (Figure 4B, Supplementary Figure S3F). P2Y_13_-IR was moderate in the granule cell layer of DG as well as in the pyramidal cell layer of CA3 and the molecular and pyramidal cell layer of CA1 (Figure 4C, Supplementary Figure S3G). P2Y_14_-IR was moderate in the molecular cell layer of DG and the stratum oriens of CA3, strong in the molecular cell layer of CA1 and weak in the stratum radiatum of CA1 (Figure 4D, Supplementary Figure S3H). P2Y_14_-IR was very low in the stratum lacunosum (CA1) (Figure 4D, Supplementary Figure S3H). 

In summary, all fifteen P2 receptors except P2X4 and P2Y_1_ were at least moderately expressed in any of the hippocampal layers (Table 1). All of these 13 receptors showed a layer-specific staining pattern (Table 1). The molecular cell layer of the CA1 was most frequently stained with an intensity of moderate or higher, while the stratum lacunosum did not show a staining intensity higher than weak with any receptor (Table 1).

### 2.2. Temporal Distribution of P2-IR in the Hippocampus

#### 2.2.1. Temporal Distribution of P2X-IR 

**Figure 5:**
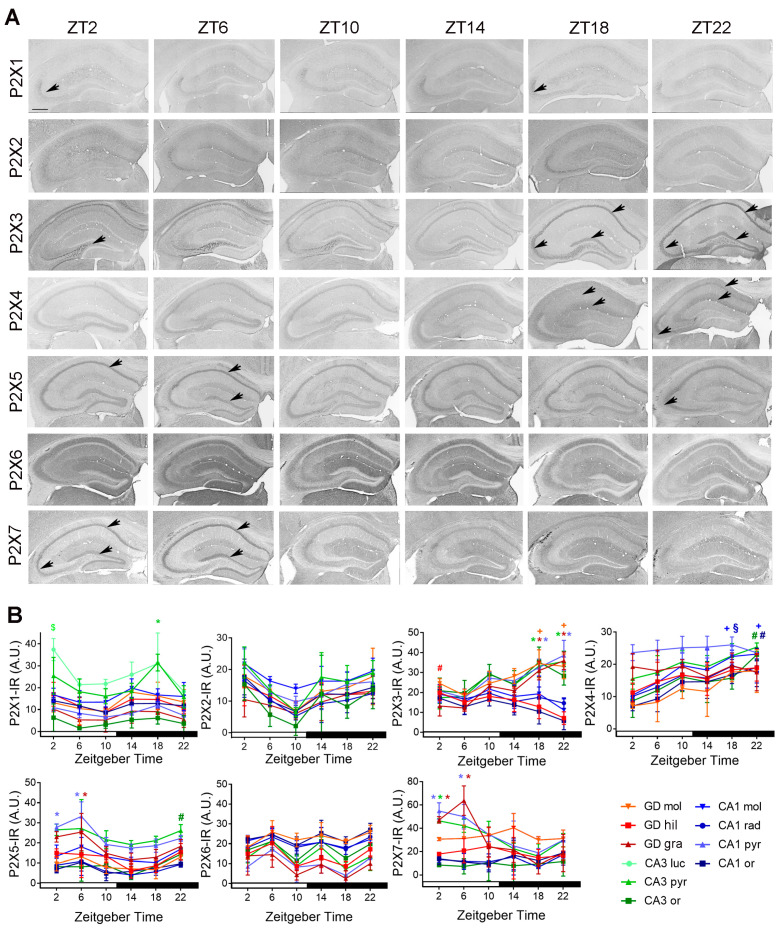
Temporal distribution of P2X-IR in the mouse hippocampus. (**A**) Representative microphotographs of P2X-IR in the hippocampus of mice killed at different zeitgeber times (ZT). Scale bar, 400 µm. (**B**) Quantitative analyses of P2X-IR in the hippocampal layers. Red, green, blue * indicate significantly (*p* < 0.05 vs. through time) elevated levels in granule (gra) and pyramidal (pyr) cell layers of dentate gyrus, CA3 and CA1, respectively. Red, green, blue **+** indicate significantly (*p* < 0.05 vs. through time) elevated levels in molecular (mol) cell layers of dentate gyrus, CA3 and CA1, respectively. Red, green, blue **#** indicate significantly (*p* < 0.05 vs. through time) elevated levels in hilus (hil) and stratum oriens (ori) of dentate gyrus, CA3 and CA1, respectively. Red, green, blue **§** indicate significantly (*p* < 0.05 vs. through time) elevated levels in stratum radiatum (rad) of CA1. Green $ indicates a significantly (*p* < 0.05 vs. through time) elevated level in stratum lucidum (luc) of CA3. Shown are the mean +/− arbitrary units (A.U.) of grey values above background of *n* = 3 mice per time point. Arrows indicate subregions of significantly high expression.

P2X1-IR was significantly increased in the pyramidal cell layer of CA3 at ZT18 as compared to ZT10 (*p* = 0.036) and ZT22 (*p* = 0.032) and in the stratum lucidum at ZT2 as compared to ZT 22 (*p* = 0.037). P2X2-IR did not show a time of day-dependent variation in any hippocampal layer. The pattern of P2X3-IR was substantially different among the mice sacrificed at different time points. In mice sacrificed during the day and the early night, P2X3-IR showed a mossy fiber-like staining and consequently an increased intensity in the hilus of the dentate gyrus (ZT2 vs. ZT22, *p* = 0.043). Whereas in mice sacrificed at mid- and late night, the staining was increased in the granule cell layer of dentate gyrus (ZT18 vs. ZT02, *p* = 0.0005; ZT18 vs. ZT06, *p* = 0.0003; ZT22 vs. ZT02 and ZT06, *p* < 0.0001) as well as the pyramidal cell layers of CA1 (ZT18 vs. ZT 02, *p* = 0.025; ZT18 vs. ZT6, *p* = 0.0044; ZT22 vs. ZT2, *p* = 0.0011; ZT22 vs. ZT06, *p* = 0.0002) and CA3 (ZT18 vs ZT 02, *p* = 0.0015; ZT18 vs. ZT06, *p* = 0.0022; ZT22 vs. ZT02, *p* = 0.0004; ZT22 vs. ZT06, *p* = 0.0006) (Figure 5A,B).

P2X4-IR was significantly increased in the molecular cell layer of the CA1 during the mid and late dark phase (ZT18 vs. ZT2, *p* = 0.013; ZT22 vs. ZT2, *p* = 0.008) at ZT18 in the stratum radiatum of CA1 (vs. ZT2, *p* = 0.013) and at ZT22 in the stratum oriens of CA1 (vs. ZT2, *p* = 0.028) and CA3 (vs. ZT2, *p* = 0.015). P2X5-IR was significant increased at ZT6 in the granule cell layer of dentate gyrus (vs. ZT 14, *p* = 0.04), as well as at ZT2 and ZT6 in the pyramidal cell layer of CA1 (ZT02 vs. ZT14, *p* = 0.048; ZT6 vs. ZT10, *p* = 0.0069; vs. ZT14, *p* = 0.0026; vs. ZT18, *p* = 0.0049; vs. ZT22, *p* = 0.035). P2X6-IR did not show a time of day-dependent variation in any hippocampal layer. P2X7-IR was significantly increased at ZT2 and ZT6 in the granule cell layer of dentate gyrus (ZT2 vs. ZT14, *p* = 0.021; ZT6 vs. ZT10, *p* = 0.017; vs. ZT14, *p* = 0.011; vs. ZT18, *p* = 0.0014; vs. ZT22, *p* = 0.0071), as well as in the pyramidal cell layer of CA1 (ZT2 vs. ZT14, *p* = 0.042; vs. ZT18, *p* = 0.0128; ZT6 vs. ZT18, *p* = 0.033) and at ZT2 in pyramidal cell layer of CA3 (vs. ZT18, *p* = 0.039) (Figure 5A,B). 

#### 2.2.2. Temporal Distribution of P2Y-IR 

**Figure 6:**
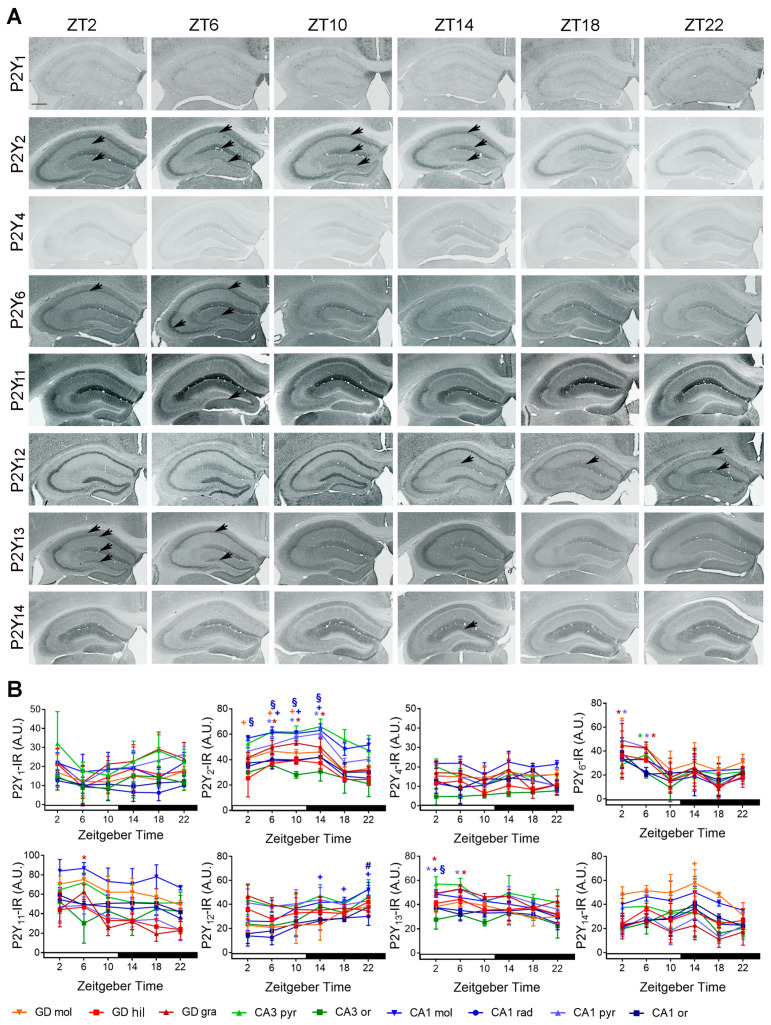
Temporal distribution of P2Y-IR in the mouse hippocampus. (**A**) Representative microphotographs of P2Y-IR in the hippocampus of mice killed at different zeitgeber times (ZT). Scale bar, 400 µm. (**B**) Quantitative analyses of P2Y-IR in the hippocampal layers. Red, green, blue * indicate significantly (*p* < 0.05 vs. through time) elevated levels in granule (gra) and pyramidal (pyr) cell layers of dentate gyrus, CA3 and CA1, respectively. Red, green, blue + indicate significantly (*p* < 0.05 vs. through time) elevated levels in molecular (mol) cell layers of dentate gyrus, CA3 and CA1, respectively. Red, green, blue **#** indicate significantly (*p* < 0.05 vs. through time) elevated levels in hilus (hil) and stratum oriens (ori) of dentate gyrus, CA3 and CA1, respectively. Red, green, blue **§** indicate significantly (*p* < 0.05 vs. through time) elevated levels in stratum radiatum (rad) of CA1. Shown are the mean +/− arbitrary units (A.U.) of grey values above background of *n* = 3 mice per time point. Arrows indicate subregions of significantly high expression.

P2Y_1_-IR did not show a time of day-dependent variation in any of the hippocampal layers. P2Y_2_-IR was significantly higher during the day and early night in dentate gyrus, granular cell layer (ZT06 vs. ZT18, *p* = 0.0013; vs. ZT22, *p* = 0.002; ZT10 vs. ZT18, *p* = 0.0003, vs. ZT22 0.0004; ZT14 vs. ZT18, *p* = 0.0013; vs. ZT22, *p* = 0.002) and molecular cell layer (ZT02 vs. ZT18, *p* = 0.026; ZT06 vs. ZT18, *p* = 0.0011; vs. ZT22, *p* = 0.0046; ZT10 vs. ZT18, *p* = 0.0026, vs. ZT22, *p* = 0.012; ZT14 vs. ZT18, *p* = 0.0015; vs. ZT22, *p* = 0.0068) as well as in CA1 molecular cell layer (ZT6 vs. ZT18, *p* = 0.021; ZT10 vs. ZT18, *p* = 0.029, ZT14 vs. ZT18, *p* = 0.0091; vs. ZT22, *p* = 0.048) and stratum radiatum (ZT02 vs. ZT18, *p* = 0.045; vs. ZT22, *p* = 0.013; ZT06 vs. ZT18, *p* = 0.0007; vs. ZT22, *p* = 0.0003; ZT10 vs. ZT18, *p* = 0.0012, vs. ZT22 *p* = 0.0004; ZT14 vs. ZT18, *p* = 0.0003; vs. ZT22, *p* < 0.0001) (Figure 6A,B).

P2Y_4_-IR did not show a time-of-day-dependent variation in any of the hippocampal layers. P2Y_6_-IR was significantly increased at ZT02 and ZT06 in granule cell layer of dentate gyrus (ZT02 vs. ZT18, *p* = 0.033; ZT06 vs. ZT18, *p* = 0.046) at ZT06 in pyramidal cell layer of CA3 (vs. ZT10, *p* = 0.0042; vs. ZT22, *p* = 0.025) and at ZT02 and ZT06 in pyramidal cell layer of CA1 (ZT02 vs. ZT18, *p* = 0.015; ZT06 vs. ZT18, *p* = 0.047). P2Y_11_-IR was significantly increased at ZT06 in granule cell layer of dentate gyrus (vs. ZT10, *p* = 0.0055; vs. ZT14, *p* = 0.0217; vs. ZT18, *p* = 0.0016; vs. ZT22, *p* = 0.0033). P2Y_12_-IR was significantly higher during the night in CA1 stratum moleculare (ZT14 vs. ZT2, *p* = 0.03, ZT18 vs. ZT2, *p* = 0.028; ZT22 vs. ZT2, *p* = 0.0006, vs. ZT10, *p* = 0.03) and at ZT22 in CA1 stratum oriens (vs. ZT2, *p* = 0.002; vs. ZT6, *p* = 0.005). P2Y_13_-IR was significantly increased at ZT2 and ZT6 in granule cell layer of dentate gyrus (ZT2 vs. ZT18, *p* = 0.016; ZT6 vs. ZT18, *p* = 0.002), as well as at ZT2 and ZT6 in CA1 pyramidal cell layer (ZT2 vs. ZT22, *p* = 0.038; ZT6 vs. ZT22, *p* = 0.0068) and at ZT2 in CA1 stratum radiatum (vs. ZT22, *p* = 0.049). P2Y_14_-IR showed a significant increase at ZT14 in the molecular cell layer of dentate gyrus (vs. ZT22, *p* = 0.04) (Figure 6A,B).

In summary, all hippocampal layers except the molecular cell layer of CA3 showed a time of day-dependent variation of at least one P2 receptor (Table 2). All P2 receptors except P2X2, P2X6, P2Y_1_ and P2Y_4_ showed a time-of-day-dependent expression in any of the hippocampal layers (Table 2). The granule cell layer of the dentate gyrus and the pyramidal cell layer of the CA1 showed a time-of-day-dependent variation for most P2 receptors which was overlapping except for P2Y_11_ (Table 2). The temporal distribution of P2 receptors in the hippocampal layers can be segregated into two large time domains, the early to mid-day (ZT02-06) and the mid to late night (ZT18-22) (Table 3). P2X5, P2X7, P2Y_2_, P2Y_6_, P2Y_11_, and P2Y_13_ could be allocated to the early to mid-day domain. P2X4, P2X5, P2Y_12_ could be allocated to the mid to late night domain. P2X1 and P2X3 changed the spatial distribution between the two time domains. P2Y_14_ did not fell into the two large time domains (increased at early night).

## 3. Discussion

Purinergic signaling plays an important role in modulating synaptic plasticity in the hippocampus neuronal network and thus in learning and memory formation. This study provides a systematic spatial and temporal mapping of fifteen P2 receptors in the mouse hippocampus as an important basis for understanding their respective potential modulatory role in hippocampal synaptic transmission. C57Bl/6J mice were used, as this is the most commonly used laboratory animal for genetic engineering techniques and preclinical studies. 

The hippocampus formation integrates cognitive and non-cognitive information for learning and memory. The long-range synaptic inputs employ different neurotransmitters and terminate at distinct layers in the dentate gyrus, the CA3 or the CA1. P2 receptors modulate synaptic transmission at the pre- and postsynaptic level. The ionotropic P2X receptors are involved in synaptic plasticity and fast interneuronal as well as neuron-glia transmission, while the metabotropic P2Y receptors are related to more long-lasting and trophic functions (reviewed by [40,41]). P2X and P2Y receptors are widely expressed by neurons and glial cells. While glial cell express most of purinergic receptors, several purinergic receptors are also identified in neuronal cells e.g., P2X2, P2X3, P2X4. P2X7. All the P2Y receptors are differentially expressed in several types of neurons [41]. Typically, P2Y_1_, P2Y_2_, P2Y_4_ and P2Y_6_ are coupled to Gq proteins whereas P2Y_12_, P2Y_13_, and P2Y_14_ are coupled to Gi proteins, resulting in the activation and reduction of the second messengers of phospholipase C and cAMP, respectively [41]. Thus, the spatial distribution of P2 receptors in the distinct hippocampal regions and layers provides important information for their potential role in modulating hippocampal function. 

The entorhinal cortex, the major interface between the neocortex and the hippocampal formation, provides important information for declarative memory, navigation and the perception of time [42]. The entorhinal cortex projects via the glutamatergic perforant path predominantly to the molecular cell layers of all fields of the hippocampus formation including the dentate gyrus, all CA fields, and the subiculum [26]. There are two major projections of perforant path fibers: (1) the entorhinal-hippocampal trisynaptic circuit from the dentate gyrus via the mossy fibers and the CA3 Schaffer collaterals to the stratum radiatum of the CA1, and (2) the projection to CA1 and the subiculum via the temporo-ammonic pathway which might be important for continuous comparison between new inputs and temporarily stored information [43]. Our data on the distribution pattern suggest slightly different roles of P2 receptors in these two projections. 

The trisynaptic pathway is especially important for episodic and spatial memory [44]. In the molecular cell layer of the dentate gyrus, the staining intensity was moderate or higher for P2X2, P2X6, P2Y_6_, and P2Y_11_, consistent with colocalization of these receptors in rat hippocampal glutamatergic nerve terminals [45]. P2X3-Ir shows a mossy fiber like appearance, which is consistent with the distribution pattern of the *P2X3* mRNA in the rat hippocampus and its suggested role in mediating fast excitatory synaptic transmission by zinc-sensitive ATP-gated channels on CA3 pyramidal cells [46]. This is also consistent with the capacity of microglia-derived ATP to modulate synaptic transmission and short-term plasticity at the mossy fiber synapses [47]. However, the study by George et al. [47] showed a strong P2X4-IR in the mossy fibers of juvenile (3 weeks old) mice. In our study using adult (12-15 weeks old) mice, P2X4-IR was not mossy fiber-like but very weak in the stratum lucidum. This inconsistency might suggest a shift in P2XR expression in the mossy fiber tract during adolescence. P2X1-IR was strong in the molecular cell layer and the stratum lucidum of the CA3, consistent with a strong post-synaptic expression in the rat hippocampus [37]. Thus, P2X3 and P2X1 might modulate the mossy fiber synapse at the pre- and postsynaptic level. A moderate P2X2-, P2X6-, P2Y_6_-, and P2Y_11_-IR could be detected in the stratum radiatum of the CA1 region, thus these receptors could potentially modulate Schaffer collateral synaptic transmission. 

The temporo-ammonic path mediates spatial memory consolidation [48], and has been implicated in chronic stress-induced depression [49] and epileptogenesis [50]. In the molecular cell layer of the CA1, the staining intensity was moderate or higher for P2X1, P2X2, P2X5, P2X6, and P2X7 as well as in P2Y_2_, P2Y_4_, P2Y_6_, P2Y_11_, P2Y_13_, and P2Y_14_. Thus, purine and pyrimidine-analogues could modulate fast and long-lasting effects of temporo-ammonic transmission via these receptors. This is consistent with our earlier study showing a role of P2Y_2_ in *p*-Akt expression and spatial working memory [51]. Moreover, ATP analogs biphasically modulate evoked glutamate release from rat hippocampal nerve terminals, the facilitation being mediated by P2X1, P2X2, and P2X3, and the inhibition by P2Y_1_, P2Y_2_, and or P2Y_4_ [45]. Thus, these receptors might play a role in fine-tuning of glutamate release from the temporo-ammonic terminals. 

In the stratum lacunosum, which receives its main long-range serotonergic and noradrenergic input from the brain stem, the staining intensity of all P2 receptors was weak. In the stratum oriens of the CA1, which receives its main long-range input from the cholinergic septal area, the staining intensity was moderate or higher for P2X2, P2X3, P2X6, P2Y_2_, P2Y_6_, and P2Y_11_. This is consistent with an interconnection of P2X2 and P2X3 with nicotinergic acetylcholine receptors at the presynaptic level to control excessive noradrenergic terminal activation in the rat hippocampus [37].

P2X2, P2X4, P2X5, P2X7, P2Y_2_, and P2Y_12_ showed a homogenous distribution in the granule cell layer of the dentate gyrus and the pyramidal cell layer of CA1 and CA3. This is consistent with earlier studies on spatial distribution of P2 expression in nocturnal rodents [33,52,53] and consistent with a general role of these P2Rs in postsynaptic modulation of synaptic strength and plasticity [40,54] in hippocampal granule and pyramidal neurons. Interestingly, the IR for P2X1, P2X6, and P2Y_11_ was stronger in the pyramidal cell layer of the CA3 region as compared to CA1, suggesting an explicit postsynaptic role of these P2Rs in CA3 pyramidal neurons. P2Y_1_-IR was considerably low in the entire hippocampus formation as compared to adjacent neocortical regions. Only few dispersed cells with interneuron-like morphology were P2Y_1_-IR. This is consistent with a P2Y_1_-mediated effect on hippocampal interneurons [55]. In the hilus, cells with interneuron-like morphology were P2X5-, and P2Y_6_,_12_ and P2Y_13_-immunoreactive. 

Hippocampal synaptic plasticity shows a time-of-day-dependent variation which are controlled by the SCN and zeitgebers such as light, activity and food (reviewed by [56]). Specifically in mice, the degree of population spike enhancement was larger in LTP during night as compared to day after Schaffer-collateral stimulation and also the decay of LTP was slower during the night [57]. As synaptic plasticity is modulated by excitatory and inhibitory, pre- and postsynaptic P2 receptor signaling (reviewed by [15]), it is of high interest to study the temporal profile of P2 receptors in mouse hippocampus. Presynaptic P2X receptors are able to facilitate inhibitory GABA transmission, thus rhythmic neuronal activity might be controlled by inhibition and disinhibition. However, the spatial resolution in this study is not sufficient to distinguish between excitatory and inhibitory neurons or subcellular localization. P2X2, P2X6, P2Y_1_, and P2Y_4_ did not show a significant variation during the 24 h cycle in any of the hippocampal layers. This is consistent with a constant expression of these receptors in the SCN [19], indicating that their expression is not affected by light and/or activity state across brain regions. All hippocampal layers except molecular cell layer of CA3 showed a time-of-day-dependent variation of at least one P2 receptor. The granule cell layer of dentate gyrus and the pyramidal cell layer of CA1 showed the highest number of P2 receptors with rhythmic expression. This is consistent with the role of granule and CA1 pyramidal cells in integrating input from entorhinal cortex, subcortical and brain stem projections and thus exteroceptive as well as interoceptive information for memory formation. The high degree of overlap in P2 receptors with rhythmic expression in these two layers suggest similar regulatory mechanisms. 

The temporal distribution of P2 receptors in the hippocampal layers can be segregated into two large time domains, the early to mid-day (ZT2-6) and the mid to late night (ZT18-22). P2X3 (in granule and pyramidal cell layers), P2X4, P2X5, and P2Y_12_ were increased during the mid-late dark phase, suggesting an association with a longer period of darkness/activity. In contrast, P2X5, P2X7, P2Y_2_, P2Y_6_, P2Y_11_, (P2Y_12_ long lasting), and P2Y_13_ were increased during the early to mid-day domain. As light is a strong aversive stimulus suppressing locomotor activity in nocturnal animals, the increase of these receptors during the early to mid-day might be a consequence of the aversive stimulus and/or reduced activity. Astrocytic ATP has been shown to modulate stress and reward response presumably mainly via P2X receptors [58]. Importantly, during the light phase, levels of extracelluar ATP rise in subcortical and cortical regions, including the hippocampus, in parallel with sleep onset in rats [59]. Enzymatic degradation of ATP leads to sequential formation of ADP, which can act on P2Y_12_ and P2Y_13_ receptors, and adenosine, which can act on P1 receptors (reviewed by [40]). 

In both the hippocampus and the SCN, the phases of rhythmic extracellular ATP levels [21,22,23,24,25,26,27,28,29,30,31,32,33,34,35,36,37,38,39,40,41,42,43,44,45,46,47,48,49,50,51,52,53,54,55,56,57,58,59,60,61,62,63,64,65,66,67,68,69,70,71,72,73,74,75,76,77,78,79,80,81,82,83,84,85,86,87,88,89,90] and cFos expression [59,91], a marker for neuronal activity as well as cholinergic [92] and NMDA-dependent glutamatergic neurotransmission [93], are opposite, indicating a general negative correlation of extracellular ATP and neuronal activity. The P2Y receptors increased during the early-mid day domain have different sensitivities to purines and pyrimidines, UTP/ATP (P2Y_2_), UDP (P2Y_6_), ATP (P2Y_11_) and P2Y_13_ (ADP) while the P2Y_12_, increased during the mid-late night domain is also more sensitive for ADP (sensitivity profiles reviewed by [40]). Thus, there is no correlation with receptor sensitivity to ADP and time domain. However, P2Y_2_, which is sensitive for both ATP and UTP, shows the long-lasting increase. The temporal profile of P2Y_2_ and P2Y_6_ (increased during early-mid light phase) and P2X3, P2X4 and P2Y_12_ (increased during the mid-late dark phase) in hippocampal layers (this study) was similar as in the SCN [19], suggesting a general regulatory mechanism and might also implicate a general role of these receptors in modulating glio-/neurotransmission during these temporal domains across brain regions. However, as the temporal profile of extracellular ATP is in antiphase in hippocampus [59] and SCN [21] a general regulation of these P2 receptors by their ligands is unlikely. The temporal shift of spatial distribution of P2X1- and P2X3-IR from stratum lucidum/mossy fiber-like staining during the early day to CA3 pyramidal cell layer staining (for P2X3 additionally in granule cell layer and pyramidal cell layer in CA1) during the mid-late night, respectively, suggests a very dynamic role of these receptors in generally modulating synaptic plasticity and specifically of input into the trisynaptic hippocampal pathway during the early day domain. 

Brancaccio et al. 2017 showed that in the SCN, circadian rhythms in intracellular calcium concentration are in anti-phase between neurons and astrocytes [94]. Taking into consideration that P2 receptors play a role in mediating intracellular Ca^+2^ concentration [95,96], it is tempting to speculate that the here described temporal change in spatial distribution of P2 receptors is related to a shift in expression between neurons and astrocytes. However, the limitation of this study is a low resolution at the cellular level needed for analyses of cell type specificity. 

In conclusion, this study provides an extensive comparative mapping of the spatial and temporal profile of P2 receptors in hippocampal subregions and layers as an important basis for further studies on the temporal dynamics of P2 receptor expression in neurons and glia cell and on the significance of the early-mid day and mid-late night domains for modulating synaptic plasticity and drug efficacy by P2 mediated purinergic signaling.

## 4. Material and Methods

### 4.1. Animals and Tissue Processing

All animal procedures were approved by the North Rhine-Westphalia State Agency for Nature, Environment and Consumer Protection, Germany (case number: 84-02.04.2013.A358) and conform to international guidelines for the care and use of animals.

Male C57BL6/J mice (12–15 weeks old) were housed in single standard cages with access to food and water ad libitum. Animals were kept under standard Zeitgeber Time (ZT) conditions with a light-dark-cycle of 12h light/12h darkness (light on 6:00 a.m. = ZT00; light off 6:00 p.m. = ZT12). Spontaneous locomotor activity was continuously recorded by means of on-cage infrared detectors (mouse-E-motion, Hamburg, Germany) and analysed using Clock lab software (Actimetrics, Wilmette, IL, USA). Three mice (*n* = 3) were killed at each of the following zeitgeber time points: ZT02, ZT06, ZT10, ZT14, ZT18, and ZT22.

### 4.2. Brain Preparation, Immunohistochemistry and Timm Staining

Brain preparation and immunohistochemistry were performed as described earlier [19]. Briefly, mice were deeply anaesthetized using ketamine:xylazine (100 mg:10 mg/kg body weight) and transcardially perfused with 0.9% NaCl followed by 4% paraformaldehyde. Brains were dissected and post fixed in 4% paraformaldehyde for 24 h and cryoprotected in 20% sucrose for another 24 h. Brains were sectioned coronally (30 µm thickness) in series by use of a cryomicrotome (Reichert-Jung, Wetzlar, Germany). Slices were permeabilized with phosphate buffered saline (PBS) containing 0.2% Triton-X 100 and were incubated in 0.24% H_2_O_2_ for 30 min at room temperature (RT). After washing with PBS/0.2% Triton-X 100, slices were preincubated for 1 h with normal rabbit or goat sera. The sections were incubated with primary antibodies against different purinergic receptors ([19] (Table 4) overnight at 4 °C, in addition to parallel negative control sections (Supplementary Figure S4). Sections were rinsed again, and then incubated with the corresponding biotinylated anti-rabbit or anti-goat secondary antibodies for 2 h at RT. Slices were further incubated with Vectastain Elite ABC Kit (Vector Laboratories, Burlingame, CA, USA) for 1 h followed by an incubation in 3.3′-diaminobenzidine (Sigma-Aldrich, St. Louis, MO, USA) for 10 min. Slices were rinsed with PBS/0.2% Triton-X 100, mounted on slides, air-dried and coverslipped with Entellan (Merck Millipore, Burlington, MA, USA).

Timm-staining was performed after a slightly modified protocol described previously [60]. Briefly, sections mounted on glass slides were incubated with buffer containing Na_2_S and NaH_2_PO_4_ before fixation with 0.3% glutaraldehyde/PBS and 70% ethanol/H_2_O for 10 min each. After rinsing, section were incubated with developer solution containing citrate-monohydrate, tri-Sodiumcitrat, hydrochinon, Gummi Arabicum, AgNO for 2 h in the dark. After rinsing with tap water, slices were incubated for 1 min in 1% Na_2_S_2_O_3_/H_2_O, again rinsed with tap water, incubated for 1 min in haematoxilin, again rinsed in tap water, dipped briefly in 0.5% HCl/70% ethanol. After rinsing with tap water sections were dehydrated in 100% ethanol, followed by xylol and embedded with DPX (Permount). 

### 4.3. Image Analysis

Photomicrographs were captured under bright-field illumination using a Keyence HS all-in-one BZ9000 Microscope (Keyence, Osaka, Japan). The microscope settings, especially light intensity and exposure time, were kept constant during all image acquisitions of the same P2R subtype. Hippocampal regions and layers were identified using Franklin and Paxinos Mouse Brain Atlas. Mossy fiber projections were visualized by Timm staining (Supplementary Figure S1). For quantitative analyses, ImageJ software was used. Spatial distribution was evaluated based on pseudocolor images (Table 5), obtained by 8-bit color system LUT 6 shades mode. For quantification of temporal distribution, the region of interest (ROI) was delineated and the mean grey value was measured. The mean grey value of the background was obtained from a corpus callosum area that doesn’t show positive staining. The ROI staining intensity in arbitrary units (A.U.) was obtained by subtracting the ROI mean gray value from the background mean gray value. Three hippocampal sections from each animal per time point for each receptor subtype were analysed.

### 4.4. Statistical Analysis

The statistical analysis was performed using the statistical GraphPad Prism software (GraphPad Software Inc., San Diego, CA, USA). Data are presented as mean  ±  SEM. Differences among different time points were analyzed by one-way ANOVA followed by Tukey’s posthoc test for multiple comparison. *p* value <  0.05 was considered statistically significant.

## Figures and Tables

**Table 1:** Spatial distribution of P2 receptor expression.

	P2X							P2Y							
	1	2	3	4	5	6	7	1	2	4	6	11	12	13	14
**CA1**															
ori	+	++	++	+	+	+++	+	+	++	+	++	++	+	+	+
pyr	+	++	+	+	+++	+	+++	+	++	+	+++	+	++	++	+
rad	+	++	+	0	+	++	+	+	+	+	++	++	+	+	+
lac	+	+	0	0	+	+	+	+	+	+	+	+	+	+	0
mol	++	++	+	+	++	+++	++	+	++	++	+++	++++	+	++	+++
**CA3**															
ori	+	+	++	+	+	++	+	+	++	+	++	+	+	+	++
pyr	+	++	+	+	++	+++	++	+	+++	+	++++	+++	++	++	+
luc	+++	0	++	0	0	+	+	0	++	+	+	+	+	+	+
mol	+++	+	++	0	0	++	0	0	++	0	++	++	+	+	++
**DG**															
mol	+	++	+	+	+	+++	+	+	+	+	++	+++	+	+	++
gra	0	+	+	+	+++	+	+++	+	++	++	+++	+	++	++	0
hil	++	++	+++	+	++	++	+	+	++	+	++	++	+	+	+

The immunoreaction intensities were given as very weak (0), weak (+), moderate (++), strong (+++), very strong (++++) based on pseudocolor images from hippocampi of mice killed at the middle of the light/inactive phase (ZT06). Abbreviations: CA, cornu ammonis, DG, dentate gyrus; ori, stratum oriens; pyr, stratum pyramidale; rad, stratum radiatum; mol, stratum moleculare; gcl, stratum granulare, hil; hilus.

**Table 2:** Time-of-day- dependent P2 receptor expression in hippocampal layers.

	P2X1	2	3	4	5	6	7	P2Y1	2	4	6	11	12	13	14
**CA1**															
ori				x									x		
pyr			x		x		x		x		x			x	
rad				x					x					x	
mol				x					x				x	x	
**CA3**															
mol															
luc	x														
pyr	x		x				x				x				
ori				x	x										
**DG**															
mol			x						x						x
gra			x		x		x		x		x	x		x	
hil			x												

x indicates a time of day-dependent expression. gra, stratum granulare; hil, hilus; luc, stratum lucidum; mol, stratum moleculare; ori, stratum oriens; pyr, stratum pyramidale; rad, stratum radiatum.

**Table 3:** Temporal distribution of P2 receptor expression in the hippocampal layers.

Zeitgeber Time						
	2	6	10	14	18	22
P2X1	+				+	
P2X2						
P2X3	+				++++	++++
P2X4					++	+++
P2X5	+	++				+
P2X6						
P2X7	+++	++				
P2Y_1_						
P2Y_2_	++	+++++	+++++	++++		
P2Y_4_						
P2Y_6_	++	+++				
P2Y_11_		+				
P2Y_12_				+	+	++
P2Y_13_	++++	++				
P2Y_14_				+		

Significantly increased expression is indicated by +, the number of + indicates the number of layers showing this significant increase.

**Table 4:** List of primary and secondary antibodies.

Antibody	Company and Number	Dilution	Reference
rabbit anti-P2X1 (H-100)	Santa Cruz, sc-25692	1:75	[61,62,63,64,65,66,67]
rabbit anti-P2X2	GeneTex, GTX10266	1:500	[19]
rabbit anti-P2X3 (H-60)	Santa Cruz, sc-25694	1:50	[68,69,70,71,72,73]
goat anti-P2X4 (N-15)	Santa Cruz, sc-15187	1:50	[61,62,63,64,65]
goat anti-P2X5 (N-16)	Santa Cruz, sc-15191	1:25	[19]
rabbit anti-P2X6	LSBio, LS-C94426	1:100	[19]
goat anti-P2X7 (L-20)	Santa Cruz, sc-15200	1:50	[74,75,76,77,78]
rabbit anti-P2Y_1_	Santa Cruz, sc-20123	1:50	[79,80,81]
rabbit anti-P2Y_2_	NovusBio, NB110-39032	1:100	[19,82,83]
rabbit anti-P2Y_4_	GeneTex, GTX87199	1:750	[17]
rabbit anti-P2Y_6_	Alomone labs, APR-011	1:100	[84,85]
rabbit anti-P2Y_11_	GeneTex, GTX108241	1:300	[19,86]
rabbit anti-P2Y_12_ (P-14)	Santa Cruz, sc-27152	1:50	[79,87,88]
rabbit anti-P2Y_13_	LSBio, LS-C145104	1:250	[19]
rabbit anti-P2Y_14_	LSBio, LS-C120603	1:250	[19]
goat anti-rabbit	Vector Laboratories, BA-1000	1:500	[89]
rabbit anti-goat	Vector Laboratories, BA-5000	1:500	[90]

**Table 5:** Pseudocolor code for evaluation of spatial distribution of P2Y receptors.

Color	Intensity
yellow	absent
magenta	very weak
light blue	weak
dark blue	moderate
green	strong
red	very strong

## Data Availability

The datasets used and analysed during the current study are available from the corresponding author on reasonable request.

## Supplementary Materials

The following are available online at https://www.mdpi.com/article/10.3390/ijms22158078/s1.
