# Supplementary material for: Analysis of Spatial and Temporal Distribution of Purinergic P2 Receptors in the Mouse Hippocampus
Source: Int J Mol Sci. 2021 Jul 28;22(15):8078. doi: 10.3390/ijms22158078 (PMC8348931; doi:10.3390/ijms22158078)
Supplement: Supplementary file 1 [file ijms-22-08078-s001.zip › ijms-1262136-supplementary.pdf]

Supplementary Figures:

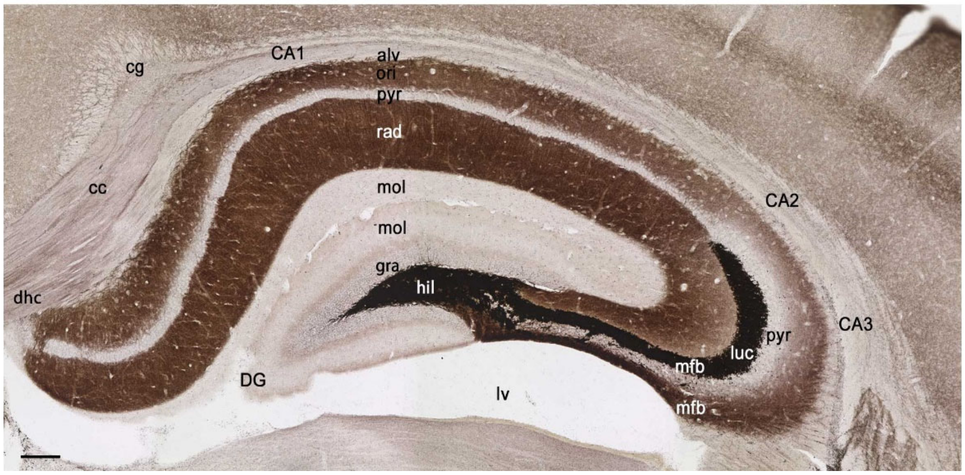

Figure S1. Representative microphotograph of mouse hippocampus with mossy fibers visualized by Timm-staining. Abbreviations: alv, alveus; CA, cornu ammonis; cc, corpus callosum; cg, cingulum; DG, dentate gyrus; dhc, dorsal hippocampal commissure; gra, stratum granulare, hil, hilus; lac, stratum lacunosum; luc, stratum lucidum; lv, lateral ventricle, mfb, mossy fiber bundle; mol, stratum moleculare; ori, stratum oriens, pyr, stratum pyramidale, rad, stratum radiatum. Scale bar = 200 μm.

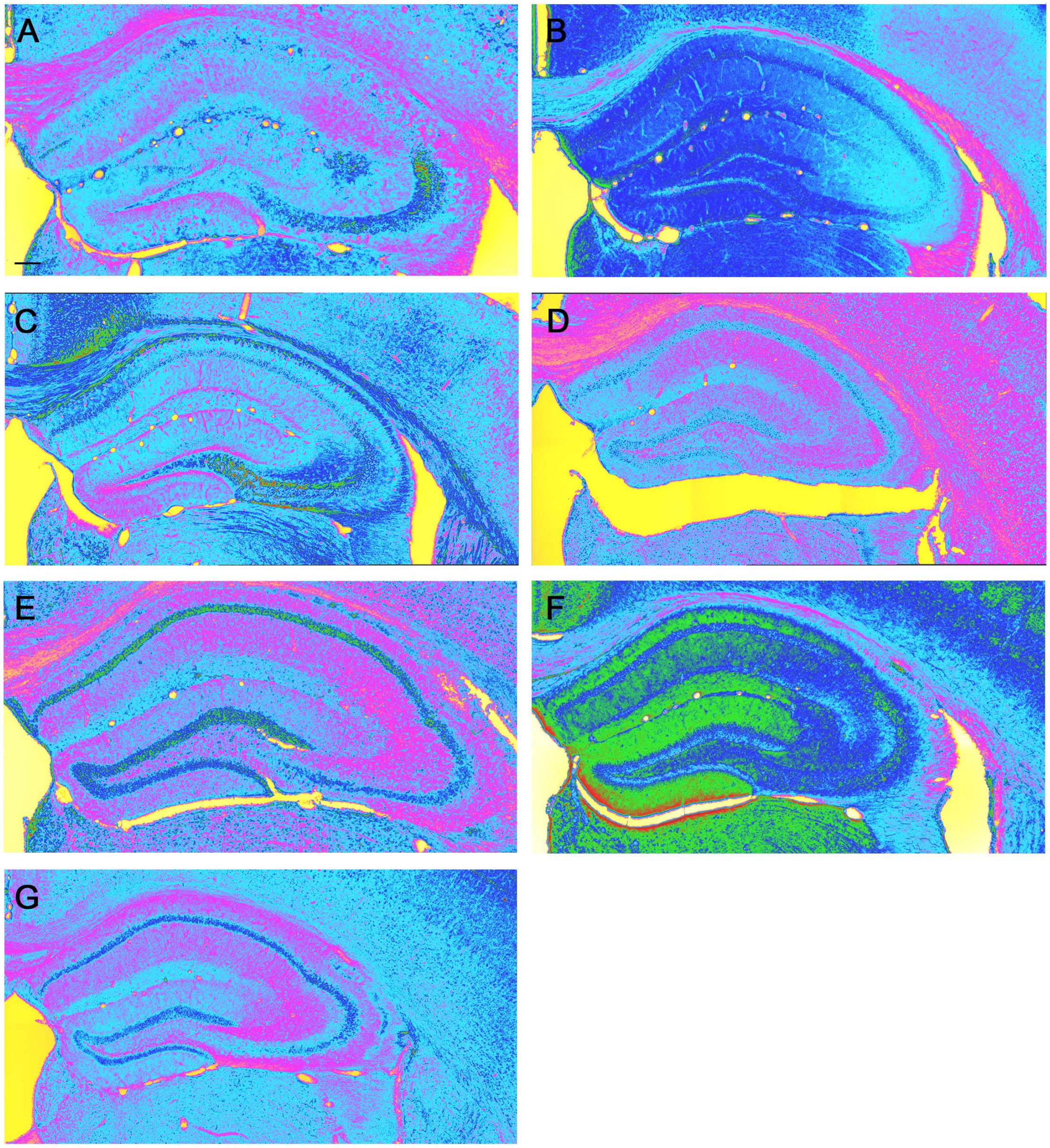

Figure S2. Representative pseudocolor images of P2X-immunoreaction in mouse hippocampus. A) P2X1, B) P2X2, C) P2X3, D) P2X4, E) P2X5 , F) P2X6 , G) P2X7 . Scale bar = 200 μm.

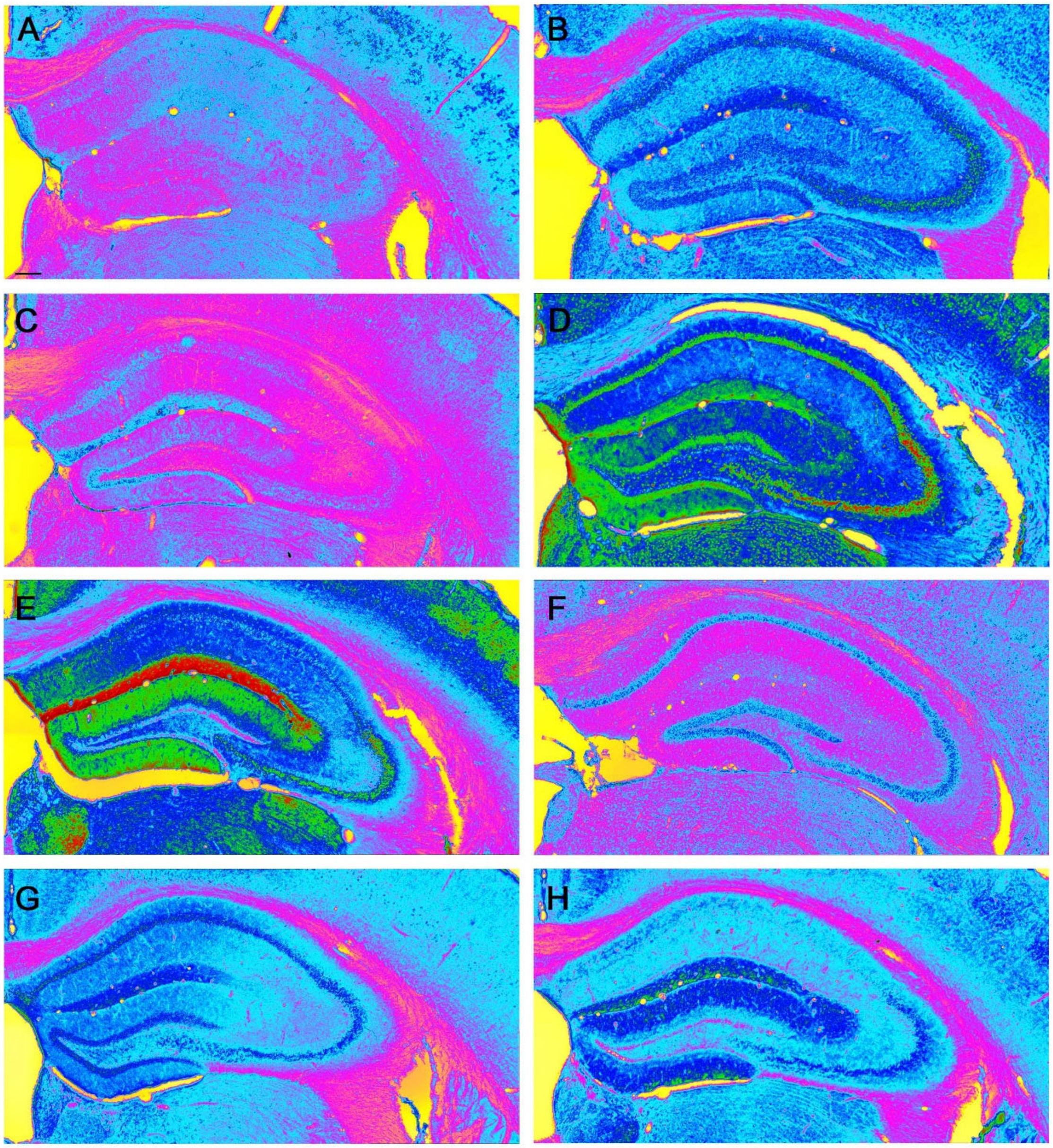

Figure S3. Representative pseudocolor images of P2Y-immunoreaction in mouse hippocampus. A) $P2Y_1$, B) $P2Y_2$, C) $P2Y_4$, D) $P2Y_6$, E) $P2Y_{11}$, F) $P2Y_{12}$, G) $P2Y_{13}$, H) $P2Y_{14}$. Scale bar = 200 μm.
